# Remyelination in animal models of multiple sclerosis: finding the elusive grail of regeneration

**DOI:** 10.3389/fnmol.2023.1207007

**Published:** 2023-06-28

**Authors:** Davin Packer, Emily E. Fresenko, Em P. Harrington

**Affiliations:** Department of Neurology, The Ohio State University College of Medicine, Columbus, OH, United States

**Keywords:** demyelination, oligodendrocyte, OPC, multiple sclerosis, remyelination

## Abstract

Remyelination biology and the therapeutic potential of restoring myelin sheaths to prevent neurodegeneration and disability in multiple sclerosis (MS) has made considerable gains over the past decade with many regeneration strategies undergoing tested in MS clinical trials. Animal models used to investigate oligodendroglial responses and regeneration of myelin vary considerably in the mechanism of demyelination, involvement of inflammatory cells, neurodegeneration and capacity for remyelination. The investigation of remyelination in the context of aging and an inflammatory environment are of considerable interest for the potential translation to progressive multiple sclerosis. Here we review how remyelination is assessed in mouse models of demyelination, differences and advantages of these models, therapeutic strategies that have emerged and current pro-remyelination clinical trials.

## Introduction

Multiple sclerosis (MS) is a chronic demyelinating, inflammatory and neurodegenerative disease of the central nervous system (CNS). Remyelination is a regenerative process by which oligodendrocytes restore myelin sheaths to demyelinated axons. Evidence from animal models indicate that remyelination can restore neuronal conduction (Smith et al., 1979, 1981), promote functional recovery (Jeffery and Blakemore, 1997; Jeffery et al., 1999; Liebetanz and Merkler, 2006; Duncan et al., 2009; Mei et al., 2016a) and protect axons from degeneration (Irvine and Blakemore, 2008; Mei et al., 2016a). Myelination and connection of the oligodendrocyte-axonal unit provides metabolic support and reciprocal signaling that promotes axonal function and survival (Nave, 2010; Simons and Nave, 2015; Thornton and Hughes, 2020; Duncan et al., 2021).

Remyelination is robust in many animal models that have been used to study remyelination, which is somewhat discordant with the heterogeneous patterns of oligodendrocyte loss, demyelination and remyelination in human pathology-based studies of MS tissue (Lucchinetti et al., 1999, 2000; Lassmann et al., 2001; Pittock and Lucchinetti, 2007). The mechanisms of remyelination failure in MS are likely complex (Franklin, 2002; Franklin and Ffrench-Constant, 2008) and may depend on lesion stage (Heß et al., 2020) and disease duration (Goldschmidt et al., 2009). Remyelination failure in human MS lesions may involve mechanisms related to oligodendrocyte apoptosis and phagocytosis (Prineas and Parratt, 2012), paucity of oligodendrocyte progenitor cells (OPCs) (Kuhlmann et al., 2008; Boyd et al., 2013; Cui et al., 2013), quiescent OPCs (Wolswijk, 1998), impaired differentiation into mature oligodendrocytes (Kuhlmann et al., 2008), and impaired contact of differentiated oligodendrocytes with demyelinated axons (Chang et al., 2002).

The inflammatory environment can modulate oligodendroglial properties including oligodendroglial survival, migration, differentiation, axon engagement and remyelination (Antel et al., 2019; Greenhalgh et al., 2020). Advances in molecular techniques have revealed transcriptional diversity in glial cell types (Zia et al., 2020; Schirmer et al., 2021) including oligodendroglia in experimental inflammatory mouse models (Falcão et al., 2018; Kirby et al., 2019; Meijer et al., 2022; Harrington et al., 2023; Hou et al., 2023) and human MS (Jäkel et al., 2019; Schirmer et al., 2019; Absinta et al., 2021), suggesting that oligodendroglia may have immunoregulatory roles in MS (Zeis et al., 2016; Harrington et al., 2020). Oligodendroglia subgroups also demonstrate distinct spatial and functional responses neuronal activity (Marisca et al., 2020) and injury (Floriddia et al., 2020) in animal models. The inflammatory environment, anatomical and subregional location, sex and age may influence the heterogeneity of oligodendroglia in MS lesions (Seeker and Williams, 2022) and could impact remyelination capacity and these factors need to be considered when modeling MS in animal models.

In animal models, aging influences oligodendroglial properties (Koutsoudaki et al., 2020; Perdaens and van Pesch, 2021; Zhang et al., 2021; Rawji et al., 2023) and progenitor-driven remyelination declines with aging (Shields et al., 1999; Sim et al., 2002) and can vary depending on regional origin of OPCs (Crawford et al., 2016). Impaired remyelination with aging can be rejuvenated with young myeloid-derived macrophages (Ruckh et al., 2012) and with fasting or metformin treatment (Neumann et al., 2019). Chronological aging is strongly associated with the development of clinical and pathological features of progressive MS (Zuo et al., 2022; Graves et al., 2023). Longer disease duration and older age is associated with higher numbers of inactive lesions and smoldering lesions (Frischer et al., 2015). Microglial activation and cortical demyelination found in progressive MS tissue (Kutzelnigg et al., 2005; Howell et al., 2011) as well as B cell follicular structures and meningeal inflammation (Howell et al., 2011) are all factors that may influence remyelination capacity in progressive MS and are not well recapitulated in mouse models.

Animal studies have revealed that newly born oligodendrocytes efficiently generate myelin sheaths (Bacmeister et al., 2020; Neely et al., 2022) and while mature surviving oligodendrocytes can generate myelin sheaths they are less efficient (Bacmeister et al., 2020; Neely et al., 2022; Mezydlo et al., 2023) and rarely restore internodes (Mezydlo et al., 2023). The differences between remyelination capacity of surviving mature oligodendrocytes and newly generated oligodendrocytes in response to demyelination likely has implications in which subsets of oligodendroglia are capable of remyelinating in human MS (Franklin et al., 2021). Radiocarbon dating with the genomic integration of ^14^C has been used to investigate the age of oligodendroglia within MS lesions and this study indicated limited production of new oligodendrocytes within shadow plaques that may have undergone remyelination suggesting that mature surviving oligodendrocytes contribute to subsequent remyelination in human MS lesions (Yeung et al., 2019). Post-mortem tissue analysis is limited by the inability to determine lesion age and extent of remyelination, and assumptions made about human oligodendrocyte progenitor properties, such as for carbon dating studies that OPCs must divide (and incorporate ^14^C) prior to differentiation despite rodent studies indicating that OPCs can directly differentiate into mature oligodendrocytes without cell division (Hughes et al., 2013). The contribution of newly born oligodendrocytes compared to surviving mature oligodendrocytes to remyelination in human MS will be difficult to determine definitively based on human post-mortem tissue analysis.

Remyelination and pro-regenerative strategies remain a major unmet need in the treatment of MS. Studying remyelination in human MS patients has been impaired by the limited array of tools to measure remyelination in humans and improved clinical measures need to be developed for incorporation into clinical trials to facilitate testing of remyelination therapies (Hill et al., 2022). While many animal models exist for investigating remyelination, this review will focus primarily on *in vivo* rodent models in which remyelination has been clearly demonstrated while highlighting limitations and advantages to consider in relation to human MS and a discussion of remyelinating therapies in clinical trials for MS.

## Identifying remyelination

**Figure 1:**
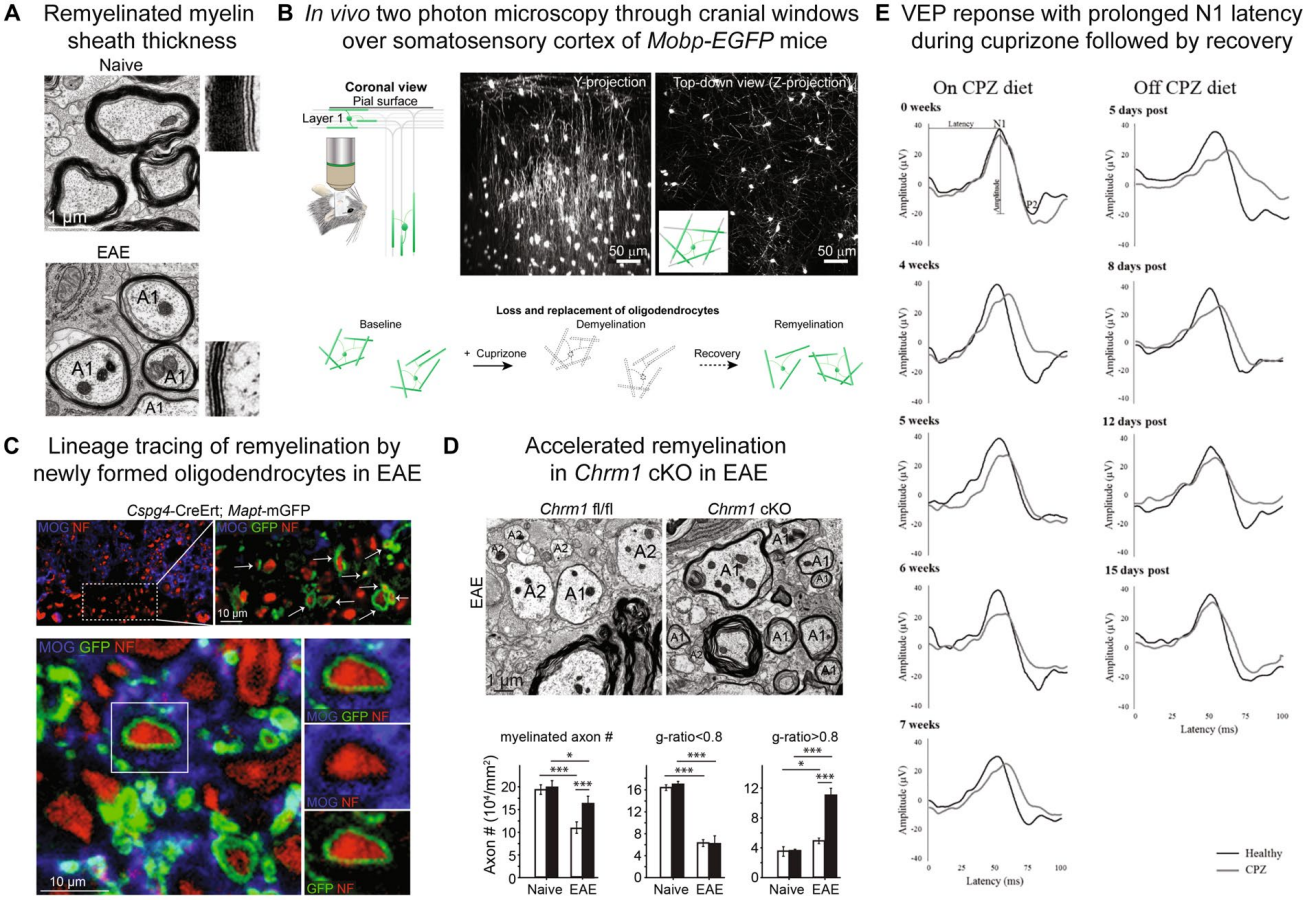
Remyelination assessment in mouse models. **(A)** Myelin sheath visualization by electron microscopy with thinner myelin sheaths in remyelinated axons (A1) in EAE compared to naïve spinal cord. **(B)** *In vivo* two photon microscopy through cranial windows over the somatosensory cortex of *Mobp-EGFP* mice allows for visualization of loss and replacement of oligodendrocytes and myelin sheaths. **(C)** Lineage tracing approach to visualize myelin sheaths from newly born oligodendrocytes utilizing *Cspg4-CreER™; Mapt-mGFP* mice and tamoxifen injection prior to EAE induction. New myelin sheaths generated by OPCs express mGFP reporter (green) and wrap neurofilament positive axons (red). **(D)** Electron microscopy of conditional knockout of muscarinic acetylcholine receptor *Chrm1* in oligodendrocytes (*Chrm1* cKO: *Cnp-Cre+; Chrm1^fl/fl^*) in EAE results in reduced axonal loss and enhanced remyelination in spinal cord indicated by significantly increased axons with g-ratios >0.8. Black bars indicate *Chrm1* cKO and white bars *Cnp-Cre-; Chrm1^fl/fl^* control. A1-remyelinated axon, A2-demyelinated axon. **(E)** Longitudinal visual evoked potential (VEP) waveforms during and off cuprizone (CPZ) diet. N1 latency is prolonged on cuprizone diet and recovers after return to normal diet. Black lines-healthy/normal diet mice, gray lines-cuprizone treated mice. **(A,C,D)** Reproduced from Mei et al. (2016a), accelerated remyelination during inflammatory demyelination prevents axonal loss and improves functional recovery, *eLife* © Creative Commons. **(B)** Reproduced from Orthmann-Murphy et al. (2020), remyelination alters the pattern of myelin in the cerebral cortex, *eLife* © Creative Commons. **(E)** Reproduced from Marenna et al. (2022), visual evoked potentials to monitor myelin cuprizone-induced functional changes, *Frontiers in Neuroscience* © Creative Commons.

Remyelination in animal models is determined through assessment of myelin sheath thickness and internode length, which is based on the early observations from animal models that remyelinated myelin sheaths are thinner than expected for axonal diameter (Blakemore, 1973, 1974; Bunge et al., 1961; Figure 1A) and internode length is shorter (Gledhill and McDonald, 1977). Myelin sheath thickness is best quantified by transmission electron microscopy (TEM) of 50–90 nm resin embedded sections and g-ratio analysis (ratio of myelinated axon to the axon alone) (Blakemore and Franklin, 2008). Remyelination of small diameter axons is difficult to determine as small axons with thinner myelin sheaths at baseline are not discernable from remyelinated axons (Stidworthy et al., 2003; Bai et al., 2016). Utilization of 3D-TEM techniques have demonstrated that reduced internodal length can be used as a readout of early remyelination in the corpus callosum, however, with time internodal length is restored to distances seen pre-demyelinating insult (Bai et al., 2016). Areas of remyelination can also be difficult to determine over time as myelin sheath thickness and remodeling closely resembles the surrounding normal appearing white matter (Neumann et al., 2020). Longitudinal intravital microscopy of oligodendroglia (Hughes et al., 2018; El Waly et al., 2020; Orthmann-Murphy et al., 2020; Bottes and Jessberger, 2021; Call and Bergles, 2021; Figure 1B) and genetic labeling strategies for lineage tracing of oligodendrocyte progenitors and subsequent myelin sheath generation (Mei et al., 2016a; Figure 1C) are alternative methods that have been utilized for assessment of remyelination and dynamics and patterns of myelin sheath formation. The complexities of accurately assessing remyelination need to be carefully considered when using animal models.

## Toxin-mediated demyelination models

Experimental models of demyelination based on the use of toxins, while these models may not recapitulate the autoimmune pathobiology of MS, they offer the advantage of stereotyped demyelination and remyelination process which has been invaluable in the investigation of the molecular mechanisms involved in remyelination.

### Focal toxins-lysolecithin and ethidium bromide

Focal demyelinating agents can be injected into the spinal cord dorsal and ventrolateral funiculi, caudal cerebellar peduncle, corpus callosum, optic nerve and subcortical white matter to create focal demyelinated lesions. Focal toxin models offer the advantage of a synchronized short demyelinating process compared to systemic toxins such as cuprizone in which demyelination is protracted and occurring in an environment that may be influenced by continued toxin exposure.

Lysolecithin or lysophosphatidylcholine (LPC) is an endogenous lysophospholipid that can be used to generate a focal demyelinating lesion by injection into white matter tracts and demyelination results through disruption of oligodendroglial cell membranes (Hall, 1972; Plemel et al., 2018) leading to oligodendroglial cell loss (McKay et al., 1998; Plemel et al., 2018). Lysolecithin-mediated demyelination is not selective for oligodendroglia. Astrocyte loss and markers of axonal injury are notable within the lesion core (Plemel et al., 2018). Generation of larger lesions or poor surgical technique can result in axonal degeneration (Blakemore and Franklin, 2008). In young animals remyelination occurs rapidly and the majority of axons are remyelinated by oligodendrocytes and Schwann cells derived from OPCs (Zawadzka et al., 2010). After the primary myelinopathy injury a secondary inflammatory response occurs which is characterized by microglial and macrophage infiltration and activation and reactive astrogliosis (Plemel et al., 2018). T cells also appear to play a role in lysolecithin-induced remyelination with Rag1 knockout and depletion of CD4 and CD8 T cells demonstrating impaired remyelination (Bieber et al., 2003).

In the lysolecithin model, myelin (Kotter et al., 2006) and extracellular matrix components such as chondroitin sulfate proteoglycans (CSPGs) (Lau et al., 2012) within the lesion can impair oligodendrocyte differentiation. Infiltrating macrophages play an important role in influencing OPC recruitment into lesions (Kotter et al., 2005) and depletion of macrophages during early stages of remyelination impairs remyelination (Kotter et al., 2001). Oligodendroglia in remyelinating lesions express transforming growth factor (TGF) β member activin-A and activin-A released from inflammatory microglia and macrophages may promote oligodendrocyte differentiation and remyelination (Miron et al., 2013).

Ethidium bromide, a DNA-intercalating agent, injected into white matter tracts creates a focal demyelinating lesion with a larger area of demyelination compared to lysolecithin (Blakemore and Franklin, 2008). Ethidium bromide, opposed to a primary myelinopathy observed in lysolecithin, is directly cytotoxic and induces cell death of oligodendrocytes and astrocytes (Blakemore, 1982). Vacuoles and splitting within the myelin sheath lamellae can occur (Yajima and Suzuki, 1979). Schwann cells have a notable contribution to remyelination in ethidium bromide lesions (Blakemore, 1982; Graça and Blakemore, 1986; Reynolds and Wilkin, 1993; Woodruff and Franklin, 1999). Ethidium bromide injections in the rat cervical spinal cord have been useful in functional studies assessing the role of remyelination on neuronal function (Jeffery and Blakemore, 1997) and cerebellar peduncle injections have facilitated investigation of repeated demyelinating events within the same site (Penderis et al., 2003).

Combining X-irradiation with ethidium bromide injection has allowed for the investigation of transplanted progenitor cells in a lesion environment devoid of endogenous remyelination potential (Blakemore et al., 2002). Neonatal OPCs (Blakemore et al., 2002), adult OPCs (Talbott et al., 2006) and adult neural stem/progenitor cells (Mothe and Tator, 2008) transplanted into ethidium bromide/X-irradiated lesions can differentiate into mature oligodendrocytes and Schwann cells capable of remyelination (Talbott et al., 2006; Mothe and Tator, 2008).

Aged animals exhibit slower remyelination after lysolecithin and ethidium bromide injection (Shields et al., 1999) and aged animals have impaired myelin debris clearance within lesions (Graça and Blakemore, 1986; Ruckh et al., 2012; Natrajan et al., 2015; Cantuti-Castelvetri et al., 2018). Agonism of retinoid X receptor gamma (RXRγ) signaling promotes macrophage clearance of myelin debris and improves remyelination efficiency in aged animals (Huang et al., 2011; Natrajan et al., 2015). Parabiotic recruitment of young monocytes (Ruckh et al., 2012) and caloric restriction and fasting mimetic metformin (Neumann et al., 2019) can accelerate remyelination in aged animals. Extracellular matrix stiffening with aging can also impair OPC differentiation and remyelination in focal toxin models (Segel et al., 2019).

### Systemic toxins-cuprizone

Cuprizone, bis-cyclohexanone-oxaldihydrazone, was first used as an animal model of demyelination in the 1960s (Carlton, 1966, 1969; Carlton, 1967). Cuprizone ingestion results in demyelination of white matter tracts including the corpus callosum, thalamus, anterior commissure and cerebellar peduncles as well as cortical gray matter (Blakemore, 1972). The mechanisms of cuprizone-induced demyelination are complex and multifactorial with evidence for contributions from primary oligodendrocyte cell death due to mitochondrial dysfunction and reactive oxygen species, oligodendrocyte cell death from toxic factors released by microglia and astrocytes, and direct attack of oligodendrocytes by innate immune cells (Matsushima and Morell, 2001; Kipp et al., 2009; Praet et al., 2014; Zirngibl et al., 2022). Cuprizone can be administered for acute and chronic durations, with 3 weeks commonly used for intravital microscopy studies (Bacmeister et al., 2020; Orthmann-Murphy et al., 2020), 4–6 weeks for acute demyelination histological studies and 12 weeks or longer for chronic demyelination studies (Kipp et al., 2009; Zirngibl et al., 2022). Oligodendrocyte apoptosis, microglial activation and reactive astrogliosis occur within the first 2 weeks of cuprizone ingestion (Buschmann et al., 2012; Wergeland et al., 2012; Zhan et al., 2020) and regional variability in degree of microglial and astrocyte activation (Gudi et al., 2009; Goldberg et al., 2015) may influence demyelination and oligodendrocyte responses. Regional differences are also seen in the extent of demyelination and oligodendrocyte loss (Gudi et al., 2009; Yang et al., 2009; Wergeland et al., 2012; Schmidt et al., 2013; Hochstrasser et al., 2019; Zhan et al., 2020), OPC proliferation (Gudi et al., 2009) and remyelination (Stidworthy et al., 2003). Axonal spheroids are present after acute cuprizone exposure (Goldberg et al., 2015) and notable axonal degeneration occurs with chronic cuprizone exposure and persists even after the remyelination period (Lindner et al., 2009). Axonal degeneration varies with cuprizone concentration, mouse strain and age (Irvine and Blakemore, 2006). Acute single and repeated cuprizone exposure both result in late onset locomotor dysfunction, brain atrophy and callosal axonal loss despite remyelination (Manrique-Hoyos et al., 2012).

Myelin loss occurs after several weeks of cuprizone ingestion and peaks at 4–5 weeks (Hiremath et al., 1998; Matsushima and Morell, 2001). One of the challenges of cuprizone-mediated demyelination is early OPC proliferative response during cuprizone ingestion resulting in endogenous remyelination even in the presence of cuprizone (Mason et al., 2000; Matsushima and Morell, 2001; Gudi et al., 2009; Zirngibl et al., 2022). Combining a rapamycin, a mammalian target of rapamycin (mTOR) inhibitor, with cuprizone ingestion can suppress spontaneous remyelination during cuprizone treatment (Sachs et al., 2014; Bai et al., 2016).

Manipulation of astrocyte and microglial responses after cuprizone-mediated demyelination can influence remyelination. Ablation of astrocytes after chronic cuprizone treatment results in improved oligodendrocyte density, remyelination and motor functional outcomes (Madadi et al., 2019). Astrocyte secretion of cytokines such as tumor necrosis factor alpha and lymphotoxins can influence demyelination and remyelination (Arnett et al., 2001; Plant et al., 2005, 2007). Astrocytes can also promote microglial accumulation and activation (Skripuletz et al., 2013). Microglial depletion reduces cuprizone-mediated demyelination and injection of colony stimulating factor 1 (CSF1) induces focal demyelination (Marzan et al., 2021) suggesting an important role for microglia in mediating demyelination during cuprizone ingestion. Microglial MER proto-oncogene tyrosine kinase (MERTK) signaling (Shen et al., 2021), triggering receptor expressed on myeloid cells 2 (TREM2) signaling (Cantoni et al., 2015; Poliani et al., 2015; Cignarella et al., 2020) and colony stimulating factor 1 (CSF1) signaling (Laflamme et al., 2018) facilitate myelin debris clearance and remyelination after cuprizone-mediated demyelination. Trem2 deficiency results in reduction of a subset of oligodendroglia that is induced in response to demyelination which may be due to delay in myelin debris clearance and induction of this subset of oligodendroglia (Hou et al., 2023).

The role of T cells in cuprizone-mediated demyelination is unclear. T cells are present in cuprizone ingestion (Remington et al., 2007) and CD8 T cells accumulate in the corpus callosum during cuprizone treatment and express activation markers (Kaddatz et al., 2021). Interleukin-17 (IL-17) secreted by T cells during cuprizone may play a role in activating microglia and mediating demyelination (Kang et al., 2012; Zimmermann et al., 2018).

Toxin models have greatly facilitated the investigation of pathways involved in oligodendroglial proliferation, recruitment, differentiation and remyelination. Promising mechanisms that are under investigation in clinical trials for remyelination in MS that have emerged from investigation of remyelination in focal and systemic toxin models include LINGO-1 antagonism (Mi et al., 2009), Nogo-A antagonism (Ineichen et al., 2017), RXR agonism (Huang et al., 2011; Natrajan et al., 2015), muscarinic receptor antagonism (Mei et al., 2014; Chen et al., 2017), semaphorin 3A (Piaton et al., 2011; Syed et al., 2011), sex hormone estrogen and testosterone supplementation (Patel et al., 2013), estrogen receptor modulators (Sicotte et al., 2007; Gonzalez et al., 2016; Rankin et al., 2019; Voskuhl et al., 2019), thyroid hormone (Bai et al., 2016; Hartley et al., 2019; Rosato-Siri et al., 2021; Pagnin et al., 2022) and metabolism modulation (Berghoff et al., 2017; Neumann et al., 2019).

The major advantages of toxin models are the robust remyelination response with stereotyped kinetics, separation of the demyelinating process from the regenerative process, minimal axonal degeneration, and decline of remyelination with aging that have allowed for discovery of targets that accelerate repair in an aged environment. While focal toxin models have these advantages, the short demyelinating insult and robust remyelination response are limitations. Diffuse CNS demyelination and white matter injury induced by cuprizone ingestion offers the advantage of an environment with prolonged oligodendroglial loss, neuronal stress and subsequent neurodegeneration, which may allow for the investigation of pathways that prevent neuronal degeneration and allow for the ability to assess motor (Manrique-Hoyos et al., 2012; Lubrich et al., 2022) and physiological outcomes (Bando et al., 2008; Cordano et al., 2022; Marenna et al., 2022). One of the potential limitations of toxin-induced demyelinating models is the absence of inflammatory niches and microenvironments found in human MS lesions such as ectopic lymphoid follicles (Negron et al., 2020) and absence of robust T and B cell responses in these toxin-mediated mouse models. However, progressive MS lesions have a paucity of inflammatory infiltrates (Frischer et al., 2009) and mechanisms involved in neurodegeneration and remyelination in progressive MS may be independent of ongoing inflammatory activity that may be best modeled in chronic demyelination models such as cuprizone with the presence of late neurodegeneration and motor deficits (Manrique-Hoyos et al., 2012). Glial heterogeneity present in toxin-mediated models (Hou et al., 2023) should be compared to glial transcriptional heterogeneity found in MS tissue (Jäkel et al., 2019; Schirmer et al., 2019; Absinta et al., 2021) to better delineate whether toxin-mediated models recapitulate glial populations present in MS lesions.

## Experimental autoimmune encephalomyelitis models

Experimental autoimmune encephalomyelitis (EAE) models are one of the most commonly used models to investigate the immunopathogenesis of MS (Gold et al., 2006; Baxter, 2007; Denic et al., 2011; Rangachari and Kuchroo, 2013; Simmons et al., 2013; Lassmann and Bradl, 2017). Immunization with emulsions of CNS tissue evolved into immunization with encephalitogenic antigens or adoptive transfer of myelin specific T cells (Baxter, 2007). EAE models can be generally classified as active (immunization of CNS peptides) or passive (adoptive transfer of encephalitogenic T cells) and they offer different advantages for the investigation of disease mechanisms of repair and remyelination.

### Active immunization

Immunization with a CNS antigen and adjuvant is used to induce active EAE in rodents and the combination of peptide and mouse strain used influences the disease course and pathology (Gold et al., 2006; Rangachari and Kuchroo, 2013; Simmons et al., 2013; Lassmann and Bradl, 2017). C57BL/6 mice were found to be susceptible to EAE with immunization with myelin oligodendrocyte glycoprotein (MOG)_35–55_ peptide (Mendel et al., 1995) which has facilitated the use of transgenic lines in investigation of disease mechanisms. In the MOG_35-55_ EAE model, demyelination is secondary to axonal injury and degeneration mediated by the adaptive and innate immune system (Kim et al., 2006; Soulika et al., 2009; Nikić et al., 2011) and does not involve a cytolytic auto-antibody response (Bourquin et al., 2003) which needs to be considered when utilizing this model for investigating mechanisms of demyelination and neurodegeneration. For modeling primary antibody-mediated demyelination in immunization models, C57BL/6 mice immunized with human MOG (Oliver et al., 2003) or rats with MOG_1-125_ peptide can be used to generate lesions with primary demyelination and axonal sparing (Storch et al., 1998). Cortical subpial demyelination can be modeled in rats with MOG immunization (Storch et al., 2006) or sub-clinical MOG immunization followed by injection of tumor necrosis factor (TNF) and IFN-γ overlying or in superficial cortical layers (Merkler et al., 2006; Gardner et al., 2013).

A limited degree of remyelination has been demonstrated in active EAE by the use of genetic lines labeling myelin sheaths generated from OPCs (*Cspg4-CreER™; Mapt-mGFP*) (Mei et al., 2016a) and transmission electron microscopy g-ratio analysis (Figures 1A,D). Significant axonal loss occurs during EAE and a large proportion of axons remain demyelinated during late EAE, 26% in spinal cord ventral white matter analyzed in this study (Mei et al., 2016a). Despite the significant degree of neurodegeneration and modest amount of remyelination, conditional knockout of M1 muscarinic receptor in oligodendrocytes (Figure 1D; Mei et al., 2016a), pharmacological antagonism of muscarinic receptors (Cordano et al., 2022) and K-opioid receptor agonism (Du et al., 2016) were able to enhance remyelination in MOG_35-55_ EAE. Visual evoked potentials can be used as biomarker for remyelination in EAE and cuprizone (Cordano et al., 2022). Immunization with proteolipid protein (PLP)_139–151_ peptide in SJL/J mice results in an acute phase of clinical disability similar to MOG_35-55_ but with partial clinical remission followed periods of fluctuating clinical scores in what has been termed “relapsing remitting” clinical course (Rangachari and Kuchroo 2013). Remyelination has been demonstrated by electronic microscopy at acute phase peak disease course with treatment with muscarinic antagonist benztropine in this EAE model (Deshmukh et al., 2013). The relapsing remitting clinical course of the SJL/J PLP_139-151_ EAE model may be useful for modeling remyelination in relapsing remitting MS however the degree of axons with high g-ratios, either demyelinated or remyelinated, normalizes during remission phase (Deshmukh et al., 2013) and whether this is due to a combination of loss of demyelinated axons or subsequent remyelination is unclear. Electrophysiological analysis of visual evoked potentials could be used as a biomarker for remyelinating in different clinical phases of the SJL/J PLP_139-151_ EAE model but ongoing inflammatory activity and neurodegeneration may limit this assessment.

Active immunization models have many features that differ from human MS pathobiology and clinical course. The robust inflammatory response and resulting secondary demyelination, early neurodegeneration (Jones et al., 2008), sparing of cerebrum, and predominance of CD4 T cells and lack of significant CD8 T cell infiltrate are all features of active EAE that do not correlate well with human MS pathobiology. The monophasic chronic disease course of MOG_35-55_ EAE does not resemble the clinical course of relapsing remitting or progressive MS. The high degree of neurodegeneration and minimal remyelination after the acute EAE phase represent challenges for the utilization of these models in assessing remyelination strategies.

### Adoptive transfer

Encephalitogenic T cells from CNS antigen immunized mice or myelin-specific T cells isolated from T cell receptor transgenic lines can be used to induce neuroinflammatory disease upon adoptive transfer into naive hosts (Paterson, 1960; Ben-Nun et al., 1981). A major advantage of adoptive transfer models is the avoidance of the priming and immune expansion phase that occurs in the periphery in immunization models that can contribute to the neuroinflammatory response. Adoptive transfer of myelin-specific CD4 T cells has been the most widely used model to generate EAE. Adoptive transfer of myelin basic protein (MBP)-specific CD8 T cells from C3H mice can induce EAE (Huseby et al., 2001). Through the utilization of transgenic lines in which CNS resident cells express foreign antigens, EAE can also be induced by adoptive transfer of CD8 T cells specific for the foreign antigen (Cabarrocas et al., 2003; Na et al., 2008; Saxena et al., 2008). These models may offer the advantage of induction of primary demyelination directly targeting oligodendrocytes (Na et al., 2008; Saxena et al., 2008) which may facilitate the exploration of mechanisms involved in CD8-mediated pathogenesis in MS, as CD8 T cells outnumber CD4 T cells in MS lesions (Booss et al., 1983; Hauser et al., 1986; Babbe et al., 2000) and CD8 infiltrates correlate with the degree of axonal degeneration in MS lesions (Bitsch et al., 2000; Kuhlmann et al., 2002).

Adoptive transfer of myelin-reactive Th17 cells after acute cuprizone ingestion has been used as a model to investigate oligodendroglial responses in the setting of T cell mediated inflammation (Baxi et al., 2015, 2017; Kirby et al., 2019) and has the advantage of perivascular inflammatory and corpus callosum infiltrates and may allow for investigation of therapeutics that target pathways activated in oligodendroglia in the context of inflammation. Whether adoptive transfer models can be used to investigate remyelination remains to be determined, but these models offer the advantage of the ability to bypass the priming phase of active EAE and investigate therapeutic effects of compounds or genetic manipulations in the absence of a priming process.

## Genetic demyelination models

Genetic models that trigger oligodendrocyte ablation or ectopic expression of interferon gamma (IFN-γ) have been used to investigate demyelination and remyelination. Initial studies of transgenic mice with targeted expression of IFN-γ from the myelin basic protein (MBP) promotor (Corbin et al., 1996) and glial acidic fibrillary protein (GFAP) promoter (LaFerla et al., 2000) demonstrated forced expression of IFN-γ during development results in CNS hypomyelination and abnormal cerebellar development. Combining GFAP promotor-driven expression of a tetracycline-controlled transactivator (tTA) (*Gfap-tTA*) with tetracycline response element (TRE) upstream of IFN-γ sequence (*TRE-IFN-γ*) in a double transgenic line allows for temporal regulation of CNS IFN-γ expression upon removal of doxycycline (Lin et al., 2004). CNS IFN-γ expression during cuprizone-mediated demyelination with removal of doxycycline during cuprizone exposure in *Gfap-tTA; TRE-IFN-γ* mice resulted in reduced differentiated oligodendrocytes and impaired remyelination (Lin et al., 2006). Prolongation of the integrated stress response (ISR) during cuprizone-mediated demyelination with CNS IFN-γ expression enhanced mature oligodendrocyte generation and remyelination (Chen et al., 2021) suggesting a beneficial role of oligodendrocyte ISR signaling in promoting remyelination.

Oligodendrocyte ablation through the combination of an inducible conditional mature oligodendrocyte Cre line (*Plp-CreER™*) with a diphtheria toxin subunit A (DTA) floxed stop reporter line (*ROSA26-eGFP-DTA*) results in oligodendrocyte apoptosis upon exposure to tamoxifen (Traka et al., 2010). Rapid oligodendrocyte loss occurs within the first week after tamoxifen injection and demyelination peaks at 5 weeks. Mice develop clinical symptoms of ataxia, tremor, hind-limb paralysis with some degree of lethality. At 10 weeks post-tamoxifen oligodendrocytes regenerate, remyelination and axonal numbers are comparable to controls, and axonal conduction assessed by spinal somatosensory evoked potentials are restored (Traka et al., 2010). Recovered animals develop a secondary fatal immune-mediated phase of demyelination that occurs around 40 weeks post-tamoxifen accompanied by focal inflammatory lesions, extensive myelin and axonal loss and the presence of MOG-specific T cells in lymphoid organs that are encephalitogenic when transferred into naïve recipients (Traka et al., 2016). This secondary immune-mediated demyelination was inhibited by tolerization with MOG_35-55_ after the initial remyelination phase. The DTA model may be useful for investigation of strategies to prevent development of a secondary adaptive immune response and investigate pathways to accelerate OPC maturation and subsequent remyelination in the setting of complete loss of mature oligodendrocytes.

Loss of function of transcription factor myelin gene regulatory factor (MYRF) that induces expression of mature myelin genes (Emery et al., 2009) in mature oligodendrocytes (*Plp-CreER™*; *Myrf^fl/fl^*) results in oligodendrocyte death, widespread demyelination, microglia and macrophage reactivity, axonal damage and incomplete remyelination (Koenning et al., 2012). Subsequent development of a secondary immune-mediated demyelination has not been reported in Myrf conditional knockout, possibly due to CNS restriction of Myrf expression to oligodendrocytes whereas Proteolipid protein 1 (Plp1) is expressed in Schwann cells which may contribute to the development of secondary autoimmunity. CNS penetrant thyroid hormone receptor mimetic, sobetirome, promoted OPC proliferation, remyelination and motor recovery in Myrf conditional knockouts (Hartley et al., 2019). While the severity of oligodendrocyte loss and demyelination in genetic oligodendrocyte ablation models may be a limitation, these models have the advantage of quantifiable motor recovery outcomes and secondary robust OPC response that can be modulated.

## Viral models

Chronic encephalomyelitis viral mouse models share pathogenic features similar to MS (Oleszak et al., 2004; Lane and Hosking, 2010; Pike et al., 2022) and may provide insight into the induction of CNS autoimmunity and pathways that are activated in CNS resident populations in response to anti-viral inflammatory responses.

### Theiler’s murine encephalomyelitis virus

Theiler’s murine encephalomyelitis virus (TMEV) is a single-stranded RNA picornavirus that causes flaccid myelitis in mice (Theiler, 1934) and can be used to induce an acute encephalomyelitis demyelinating disease through intracerebral infection. BeAn and Daniel’s TMEV strains can cause a biphasic disease process characterized by an acute infectious phase and viral clearance followed by a secondary progressive chronic demyelinating myelitis phase in susceptible mouse strains (Oleszak et al., 2004). Oligodendrocytes, astrocytes and macrophages are viral reservoirs during chronic infection (Rodriguez et al., 1983; Lipton et al., 1995) and demyelination occurs within areas of microglia and macrophage activation generating demyelinating lesions with variable axonal injury and remyelination that varies depending on mouse strain (Bieber et al., 2005). Macrophages and microglia play a critical role in immune-mediated demyelination in the TMEV model and mouse strain differences in susceptibility to demyelination may be due to differences in macrophages and microglia (Dal Canto et al., 1996). The TMEV model offers the advantage of a cytotoxic axonal injury mechanism (Rivera-Quiñones et al., 1998; Howe et al., 2007) which may facilitate the investigation of immune-mediated mechanisms of axonal injury and neurodegeneration.

### Mouse hepatitis virus

Mouse hepatitis virus (MHV) is a positive-strand RNA virus with neurotropic strains that can be used to induce a chronic demyelinating disease through intracranial or intranasal inoculation of susceptible mouse strains. An acute encephalomyelitis phase is followed by a secondary phase of demyelination and remyelination (Bender and Weiss, 2010; Lane and Hosking, 2010). MHV-specific T cells appear to instigate demyelination (Wu et al., 2000; Dandekar et al., 2001, 2004; Dandekar and Perlman, 2002). Diffuse macrophage and microglial activation and upregulation of oxidative stress pathways in the MHV model resembles changes seen in MS tissue (Schuh et al., 2014). Microglia play a critical role in the clearance of myelin debris and facilitating remyelination (Sariol et al., 2020). Oligodendrocytes that survive the acute MHV infection have prolonged MHC class I expression (Pan et al., 2020). Viral encephalomyelitis models offer the ability to investigate microglial, astrocyte and oligodendrocyte secondary responses to an inflammatory viral insult and subsequent repair mechanisms.

## Remyelination therapies-from mouse to human

High-throughput screens that evaluate the ability of compounds to promote oligodendrocyte differentiation *in vitro* have been used to identify pathways with remyelination potential (Joubert et al., 2010; Deshmukh et al., 2013; Mei et al., 2014; Najm et al., 2015; Porcu et al., 2015; Lariosa-Willingham et al., 2016; Mei et al., 2016b). Many pathways and compounds that have demonstrated improved remyelination in animal models have moved to human MS clinical trials (Table 1).

**Table 1:** Remyelinating therapies in clinical trials.

Target	Drug	Mechanism	Model	Phase	Trial ID	Inclusion criteria	Treatment protocol	Primary outcome	Results
*Axon-Oligo*	Ozanezumab	Anti-Nogo-A mAb	LPC	1	NCT01424423	RRMS or SPMS, 2 relapses past 24 months or 1 relapse or enhancing lesion in last 12 months, EDSS <5.5	Single dose Ozanezumab or placebo	Safety and tolerability of single dose	Terminated
	Opicinumab	Anti-LINGO-1 mAb	CUP, LPC	2	NCT01721161 RENEW	Acute ON-no prior MS diagnosis, first unilateral ON, within 28 days of symptom onset	Following IV steroids once every 4 weeks Opicinumab or placebo for 6 doses	24 week ff-VEP	Significantly reduced VEP latency in per-protocol group (Cadavid et al., 2017)
	Opicinumab	Anti-LINGO-1 mAb	CUP, LPC	2	NCT01864148 SYNERGY	RRMS or SPMS with evidence of disease activity in past 12 months	Once every 4 weeks Opicinumab or placebo until 72 weeks and treatment with Avonex for 84 weeks	72 week EDSS, 9HPT, T25FW, PASAT	No significant improvement in disability (Cadavid et al., 2019)
	Opicinumab	Anti-LINGO-1 mAb	CUP, LPC	2	NCT03222973	RRMS or SPMS, EDSS 2–6, MS diagnosis within past 20 years, one new lesion or relapse in past 24 months, stable dose of interferon beta, dimethyl fumarate, or Tysabri	Once every 4 weeks Opicinumab or placebo until 72–96 weeks	72 week EDSS, 9HPT, T25FW	Terminated
*Oligo*	Pepinemab (VX15/2503)	Anti-Semaphorin 4D mAb	EthBr, LPC	1	NCT01764737	MS, MS diagnosis for at least 1 year, EDSS 0–6.5	Pepinemab dose escalation or placebo	safety and tolerability	Completed (LaGanke et al., 2017)
	GSK239512	Histamine H3 receptor antagonist	CUP	2	NCT01772199	RRMS on stable dose of Avonex or Copaxone for 1 year or greater, MS diagnosis within past 10 years, EDSS 1–4.5	GSK239512 or placebo for 48 weeks	MTR change new lesion >70 days from lesion appearance	Significantly reduced mean MTR change (Schwartzbach et al., 2017)
	Clemastine	muscarinic (M1) AChR antagonist	LPC, CUP, EAE	2	NCT02040298 ReBUILD	RRMS, VEP latency delay >125 ms in at least one eye with RNFL >70 μm in that eye, no ON prior 6 months, stable DMT, MS diagnosis within past 15 years, EDSS 0–6	Crossover study Clemastine or placebo	3 month ff-VEP latency change	Significantly reduced latency (Green et al., 2017)
	Clemastine	muscarinic (M1) AChR antagonist	LPC, CUP, EAE	2	NCT0252131-ReCOVER	Acute demyelinating ON within 3 weeks from symptom onset	Clemastine or placebo for 3 months followed by re-evaluation at 9 months	9 month ff-VEP latency change and LC-VA change	Recruiting
	PIPE-307	muscarinic (M1) AChR antagonist	LPC, CUP, EAE	1	NCT04725175	Healthy subjects	PIPE-307 or placebo	Safety and tolerability	Completed
	BN201	NDRG1 phosphorylation	CUP	1	NCT03630497	Healthy subjects	BN201 or placebo	Safety and tolerability	Completed
	Liothyronine (T3)	Thyroid hormone	CUP, LPC	1	NCT02760056	MS	Liothyronine or placebo	Maximum tolerated dose	Completed (Wooliscroft et al., 2020)
	Liothyronine (T3)	Thyroid hormone	CUP, LPC	1	NCT02506751	RRMS, SPMS or PPMS, euthyroid, EDSS 3–7.5	Liothyronine dose escalation	Incidence of adverse events	Completed
*Macrophage* *Microglia*	CHS-131 (INT-131)	PPARγ modulator	EthBr, LPC	2	NCT02638038	RRMS diagnosed past 3 years or less	INT-131 or placebo	6 month new enhancing lesions	Completed
	Bexarotene (IRX4204)	RXRγ agonist	EthBr, LPC	2	ISRCTN 14,265,371 CCMR One	RRMS, on dimethyl fumarate for at least 6 months, EDSS 0–6	Bexarotene or placebo for 6 months	6 month patient-level mean lesional MTR change	Poor tolerability, no change in mean MTR, reduced VEP latency (Brown et al., 2021)
	Pioglitazone	PPARγ agonist	EthBr, LPC	1	NCT00242177	RRMS, Avonex or Rebif for 1 year or more, EDSS 1–6.5	Pioglitazone or placebo	Safety and tolerability	Completed
	rHIgM22	Human IgM promotes myelin phagocytosis	CUP, TMEV	1	NCT01803867	MS	rHIgM22 or placebo	Safety and tolerability	Completed
	rHIgM22	Human IgM promotes myelin phagocytosis	CUP, TMEV	1	NCT02398461	MS, acute relapse in last 30 days with at least one new enhancing lesion	rHIgM22 or placebo	Safety and tolerability	Completed (Greenberg et al., 2022)
*Metabolism*	Metformin	Metabolism	EthBr	1,2	NCT04121468	MS with anterior visual pathway involvement and > 6 months from ON or relapse, 10–26 years old, stable DMT, latency delay >115 ms one eye or > 10 ms difference between eyes, RNFL thickness equal or > 60 μm, EDSS 0–6	Metformin for 3–9 months	Safety and tolerability	Recruiting
	Dietary interventions	Metabolism	EthBr		NCT03508414	RRMS, last 2 years one or more relapses or one or more new lesions, stable DMT, EDSS <4.5	Ketogenic, intermittent fasting or vegetarian diet	18 month change in new T2 lesions	Active
*Hormone*	Bazedoxifene	Estrogen receptor modulator, cholesterol biosynthesis	LPC	2	NCT04002934 ReWRAP	RRMS, female sex assigned at birth 45–65 years old or 40+ years and post-menopausal, latency delay >118 ms ff-VEP at least one eye, RNFL >70 μm same eye, stable DMT, MS diagnosis within past 20 years, no optic neuritis in involved eye 10 years or more, EDSS 0–6	Bazedoxifene acetate and placebo	3 and 6 month ff-VEP latency change	Recruiting
	Estroprogestin Oral contraceptives	Estrogen receptor agonist	CUP	2	NCT00151801	RRMS, female sex assigned at birth, 18–40 years old, no estroprogestins in past 3 months, EDSS 0–4	Randomized to IFNβ1a, IFNβ1a + desogestrel 150 μg/etinilestradiol 20 μg or IFNβ1a + desogestrel 25 μg/etinilestradiol 40 μg	Safety and tolerability, relapse rate, EDSS, functional composite score	Significantly reduced cumulative unique active lesions in estroprogestins groups (Pozzilli et al., 2015)
	Estriol and progesterone	Estrogen receptor agonist	CUP			MS, female sex assigned at birth, EDSS 0–6.5, no oral contraceptives	6 month pre-treatment followed by estriol 8 mg/day for 6 months followed by 6 month post-treatment	number and volumes of T2 and enhancing lesions, EDSS, 9HPT, PASAT	Significant decrease in volume and number of enhancing lesions during estriol treatment period (Sicotte et al., 2002)
	Testosterone (Nebido)	Androgen receptor	CUP	2	NCT03910738 TOTEM RRMS	RRMS, male sex assigned at birth, hypogonadism with serum testosterone <15 nmol/L, on Tysabri, fingolimod or Ocrelizumab for 1 year or more, no relapses in prior year, EDSS 0–7	Intramuscular testosterone udecanoate (Nebido) or placebo	Thalamic atrophy and transverse diffusion	Recruiting
	Testosterone (Androgel)	Androgen receptor	CUP	1,2	NCT00405353	RRMS, male sex assigned at birth, at least one relapse prior 2 years, EDSS 0–5, significant T2 lesion burden	Pretreatment 6 months followed by 12 months of Androgel	12 month change in whole brain atrophy	Significantly reduced brain atrophy (Sicotte et al., 2007)
*Combination Therapy*	Metformin and Clemastine		LPC, CUP, EAE, EthBr	2	NCT05131828CCMR Two	RRMS, latency delay >118 ms at least one eye, EDSS 0–6, stable DMT	Metformin and Clemastine combination or placebo	26 week ff-VEP latency change	Recruiting
	Pioglitazone, Clemastine, Dantroline, Pirfenidone		LPC, CUP, EAE, EthBr	1,2	NCT03109288TRAP-MS	PPMS or SPMS with progression on CombiWISE, EDSS 1–7.5	One or two study drugs	1.5 year CombiWISE progression rate	Recruiting

Clinical trials in optic neuritis and multiple sclerosis of pathways with demonstrated enhanced remyelination in animal models. AChR, acetylcholine receptor; CUP, cuprizone; DMT, disease modifying therapy; EAE, experimental autoimmune encephalomyelitis; EDSS, expanded disability status scale; EthBr, ethidium bromide; ff-VEP, full field visual evoked potential; IFNβ1a, interferon beta 1a; LC-VA, low contrast visual acuity; LPC, lysophosphatidylcholine; mAb, monoclonal antibody; MS, multiple sclerosis; MTR, magnetization transfer ratio; NDGR1, N-myc downstream regulated gene 1; OCT, optical coherence tomography; ON, optic neuritis; PASAT, paced auditory serial addition test; PPMS, primary progressive multiple sclerosis; QOL, quality of life; RNFL, retinal nerve fiber layer; RRMS, relapsing remitting multiple sclerosis; SPMS, secondary progressive multiple sclerosis; TMEV, Theiler’s murine encephalomyelitis virus; T25FW, timed 25 foot walk; VEP, visual evoked potential; 9HPT, nine hole peg test.

Assessment of remyelination in MS clinical trials has been challenging, with few validated clinical tools to assess remyelination in humans (Hill et al., 2022). Measurement of visual pathway conduction speed with visual evoked potential (VEP) has allowed for assessment of the change in P100 latency which correlates with remyelination in autoimmune encephalomyelitis (Cordano et al., 2022) and toxin (Marenna et al., 2022) mouse models (Figure 1E). Multifocal VEP (mf-VEP) compared to full-field VEP (ff-VEP) may be a more accurate measure of remyelination after optic neuritis (Pihl-Jensen et al., 2017) and is a secondary outcome measure in NCT05131828-CCMR Two trial. Opicinumab treatment after first time acute optic neuritis significantly reduced ff-VEP latency in the per-protocol population at 32 weeks in the RENEW trial (Cadavid et al., 2017). Opicinumab combined with interferon beta-1a did not demonstrate a significant improvement in disability at 72 weeks in SYNERGY trial, however univariate analysis suggested that younger age, shorter disease duration and higher baseline brain volumes may be associated with improved disability (Cadavid et al., 2019). The ReBUILD clemastine placebo crossover trial in relapsing remitting MS (RRMS) with chronic optic neuropathy demonstrated significantly improved ff-VEP latency while on clemastine treatment (Green et al., 2017). New MRI techniques such as myelin water fraction (MWF), diffusion tensor imaging (DTI) and magnetization transfer ratio (MTR) are currently being developed to assess remyelination (Hill et al., 2022). MTR has shown promise as an imaging measure of remyelination with significant MTR lesion change in GSK239512 (Schwartzbach et al., 2017) and in gray matter and brainstem lesions in bexarotene (Brown et al., 2021). Continued development of clinical techniques to assess remyelination and thoughtful design of patient inclusion criteria and outcome measures are critical for designing clinical trials for remyelination therapies in MS. Rigorous pre-clinical testing of remyelinating pathways and compounds in animal models has facilitated the success of recent clinical trials and allowed for multiple promising therapies for myelin repair for MS.

## Conclusion

Animal models of MS with demonstrated remyelination capacity vary considerable in their mechanisms of demyelination, inflammatory infiltrates, degree of ongoing inflammatory activity, axonal loss and neurodegeneration and extent of remyelination. Focal toxin models offer the advantage of stereotyped remyelination after a short single demyelinating insult which has allowed for the investigation of factors that promote or inhibit this robust reparative response. A prolonged demyelinating insult predominated by corpus callosum and cortical demyelination and subsequent neurodegeneration and motor decline can be modelled with chronic cuprizone exposure and may share some features of the neurodegenerative process in progressive MS. Both cuprizone and EAE models induce inflammatory subsets of glia that are found in MS tissue and further investigation of how these subsets of glia contribute to ongoing inflammatory activity and promote or inhibit repair may offer insight into potential mechanisms to modulate remyelination in inflammatory settings. While no single animal model recapitulates the pathobiology of MS, considerations of the limitations and advantages of each model should be taken into account when investigating remyelination and translating animal model findings to human MS.

## Author contributions

DP and EH contributed to writing the manuscript. EF contributed to researching and generating the clinical trial table. All authors contributed to the article and approved the submitted version.

## Conflict of interest

The authors declare that the research was conducted in the absence of any commercial or financial relationships that could be construed as a potential conflict of interest.

## Publisher’s note

All claims expressed in this article are solely those of the authors and do not necessarily represent those of their affiliated organizations, or those of the publisher, the editors and the reviewers. Any product that may be evaluated in this article, or claim that may be made by its manufacturer, is not guaranteed or endorsed by the publisher.
